# Accuracy of the Sysmex UF-5000 analyzer for urinary tract infection screening and pathogen classification

**DOI:** 10.1371/journal.pone.0281118

**Published:** 2023-02-01

**Authors:** Hua Wang, Fei-Fei Han, Jian-Xun Wen, Zhi Yan, Yan-Qiu Han, Zhi-De Hu, Wen-Qi Zheng

**Affiliations:** 1 Department of Laboratory Medicine, The Affiliated Hospital of Inner Mongolia Medical University, Hohhot, China; 2 Department of Parasitology, The School of Basic Medical Sciences, Inner Mongolia Medical University, Hohhot, China; 3 Department of Medical Experiment Center, The School of Basic Medicine, Inner Mongolia Medical University, Hohhot, China; Universita Politecnica delle Marche, ITALY

## Abstract

The screening performance of urine flow cytometry parameters (e.g., white blood cell and bacteria) for urinary tract infection (UTI) has been widely recognized. The majority of previous studies, however, investigated the screening performance of Sysmex UF-1000i urine flow cytometer. This study aimed to investigate the screening performance of Sysmex UF-5000 analyzer, a third-generation urinary flow cytometer, for UTI and its novel parameter named Gram flag for discriminating gram-positive and negative pathogens. Urine specimens sent to the clinical microbiology laboratory of our hospital for bacterial culture between September 13, 2021, and November 15, 2021, were prospectively and consecutively collected. The Sysmex UF-5000 analyzer was used to determine urine white blood cell (WBC) and bacteria simultaneously. A chemical strip was used to assess urine nitrate. UTI was defined as positive urine bacterial culture > 10^4^ CFU /ml. The receiver operating characteristics (ROC) curve, nomogram, decision tree, and decision curve were used to determine the screening performance of urine WBC, nitrate, and bacterial. A total of 246 UTIs and 425 non-UTIs were enrolled. The areas under the ROC curve (AUCs) for WBC and bacterial were 0.74 and 0.86, respectively. The decision curve showed that urine bacteria had a higher benefit than WBC. The nomogram indicated that urine bacterial had the largest effect on the probability of UTI. The sensitivity and specificity of the decision tree were 0.69 and 0.95, respectively. The flag of Gram-negative had a positive predictive value (PPV) of 0.93 in patients with urine bacteria > 1367 /μl. Therefore, we conclude that urine bacteria determined by the Sysmex UF-5000 had higher screening performance and greater benefit than WBC. The decision tree can be used to improve the screening performance of routine urinary parameters. The flag of Gram-negative is a reliable indicator to confirm gram-negative bacteria infection in UTI patients.

## Introduction

Urinary tract infection (UTI) is a highly prevalent infectious disease in hospitalized patients and the general population [1, 2]. UTI patients usually visit the hospital with symptoms of dysuria, urinary frequency, and urgency [1]. These symptoms are not specific to UTI and urine bacteria culture is thus needed to verify UTI patients among suspected UTI patients. Although urine culture is the gold standard for UTI diagnostics, it has some limitations, such as a long turnaround time (TAT), labor-intensive nature, and high risk of contamination. Furthermore, a large portion of specimens sent to the clinical microbiology laboratory are culture-negative, thus increasing the workload of laboratory workers. Therefore, it is of great value to develop screening methods to verify patients at high risk of UTI. The screening methods can reduce unnecessary urine culture orders in low-risk patients, limit unnecessary antibiotics, and reduce workload burden and cost of clinical microbiology laboratory.

Routine urine parameters, such as nitrate, bacteria, and white blood cell (WBC) count, are usually used for UTI screening [3, 4]. The dipstick test determines the nitrate, while bacterial and leukocyte counts are determined by automated urine flow cytometry. The most widely used urine flow cytometry device is the Sysmex UF-1000i, and a previous meta-analysis indicated that the bacteria and WBC determined by the Sysmex UF-1000i have high performance for UTI screening [4]. The Sysmex UF-5000 is the third-generation of urine flow cytometer [5, 6]. Compared with the UF-1000i, a novel function of the UF-5000i is its ability to discriminate gram-positive and gram-negative bacteria in urine specimens [7]. This parameter is termed the Gram flag. There are three types of flag, gram-positive, gram-negative and mixed, which indicate the possible types of bacteria in urine. Some studies have been performed to evaluate the accuracy of this novel parameter, but the results vary [7–11]. In addition, some novel statistical methods such as nomogram and decision tree have rarely been used to investigate the screening performance of the Sysmex UF-5000. This study aimed to (i) evaluate the performance of the Sysmex UF-5000 for UTI screening and (ii) evaluate the accuracy of the UF-5000 for discriminating gram-positive and gram-negative bacteria.

## Materials and methods

### Patients enrollment and specimens collection

The midstream urine specimens sent to the clinical microbiology laboratory of our hospital for urine culture between September 13, 2021, and November 15, 2021, were collected. The urine specimens were collected in a sterile urine cup for urine culture first, and the remaining urine specimens were simultaneously tested by the URIT 1600 chemical analyzer (Youlite Corp, Guilin, China) and Sysmex UF-5000 (Sysmex, Kobe, Japan). The time interval between urine culture and analyses was less than 6 hours. Urine culture was used to ascertain UTI in these patients, and those with urine bacteria ≥10^4^ CFU /ml were defined as UTI patients, according to the standards of previous studies [12, 13]. Urine nitrate, WBC, bacteria, urine culture results, and the Gram flag were used for analysis. Patients aged <18 years were excluded from this study. The authors had access to information that could identify individual participants during data collection.

### Statistical analysis

The continuous variables were expressed as medians and quartiles, and the Kolmogorov-Smirnov test verified their normal distribution. The Mann-Whitney U test was used to compare continuous variables between the two studies. Categorized data were compared with the Chi-square test. Receiver operating curve (ROC) and decision curve [14] analyses were used to evaluate the screening performance and net benefit of the urine parameters. The areas under the ROC curves (AUCs) were compared by the method proposed by DeLong [15]. Spearman correlation analysis was used to study the correlation between bacteria and WBC. All analyses were performed in R (version 4.0.5), and a p value <0.05 was considered statistically significant.

### Ethics statements

This study protocol was reviewed and approved by the ethics committee of the Affiliated Hospital of Inner Mongolia Medical University (KY2021027; WZ2022015). Informed consent was waived because both the urine chemistry and sedimental analyses were performed with the remaining urine culture specimen.

## Results

### Characteristics of the participants

A total of 671 participants, 246 UTIs and 425 non-UTIs, were enrolled in this study. Their clinical characteristics are summarized in **Table 1**. Similar to previous studies [3, 10, 11, 16], increased WBC and bacteria and a positive rate of urine nitrate were observed in UTI patients (*p*<0.05 for all). The correlation coefficient between WBC and bacteria was 0.55 (*p*<0.001). Only 92 UTI patients (n = 246) were verified by nitrites, and the sensitivity of nitrites for detecting UTI was thus only 37%. But its specificity was 97%. Among 92 UTI patients with positive nitrites, 86 were infected by gram-negative bacteria.

**Table 1:** Clinical characteristics of the participants.

Variables	Total	Non-UTI	UTI	*p*
	(n = 671)	(n = 425)	(n = 246)	
**Age, years**	63 (50, 71)	61 (48, 70)	66 (55, 73)	< 0.001
**WBC, /μl**	23(6, 114)	12 (3, 40)	74 (23, 259)	< 0.001
**Bacteria, /μl**	44 (8, 761)	17 (3, 64)	4277 (126, 29095)	< 0.001
**Nitrate, n (%)**				< 0.001
**Negative**	568 (85)	414 (97)	154 (63)	
**Positive**	103 (15)	11 (3)	92 (37)	
**UTI flag, n (%)**				< 0.001
**Negative**	578 (86)	422 (99)	156 (63)	
**Positive**	93 (14)	3 (1)	90 (37)	
**Gram flag, n (%)**				< 0.001
**Negative**	148 (22)	14 (3)	134 (54)	
**Positive**	119 (18)	64 (15)	55 (22)	
**Unclassified**	404 (60)	347 (82)	57 (23)	
**Bacteria cultured, n (%)**				< 0.001
**Negative**	190 (28)	13 (3)	177 (72)	
**Positive**	40 (6)	5 (1)	35 (14)	
**Others**	43 (6)	9 (2)	34 (14)	
**Mixed**	398 (59)	398 (94)	1 (0)	

WBC, white blood cell; UTI, urinary tract infection. Continuous data were presented as median and quartile. Categorical data were presented as absolute numbers and percentages.

### Performance and net benefit of urine parameters for UTI screening

**Fig 1:**
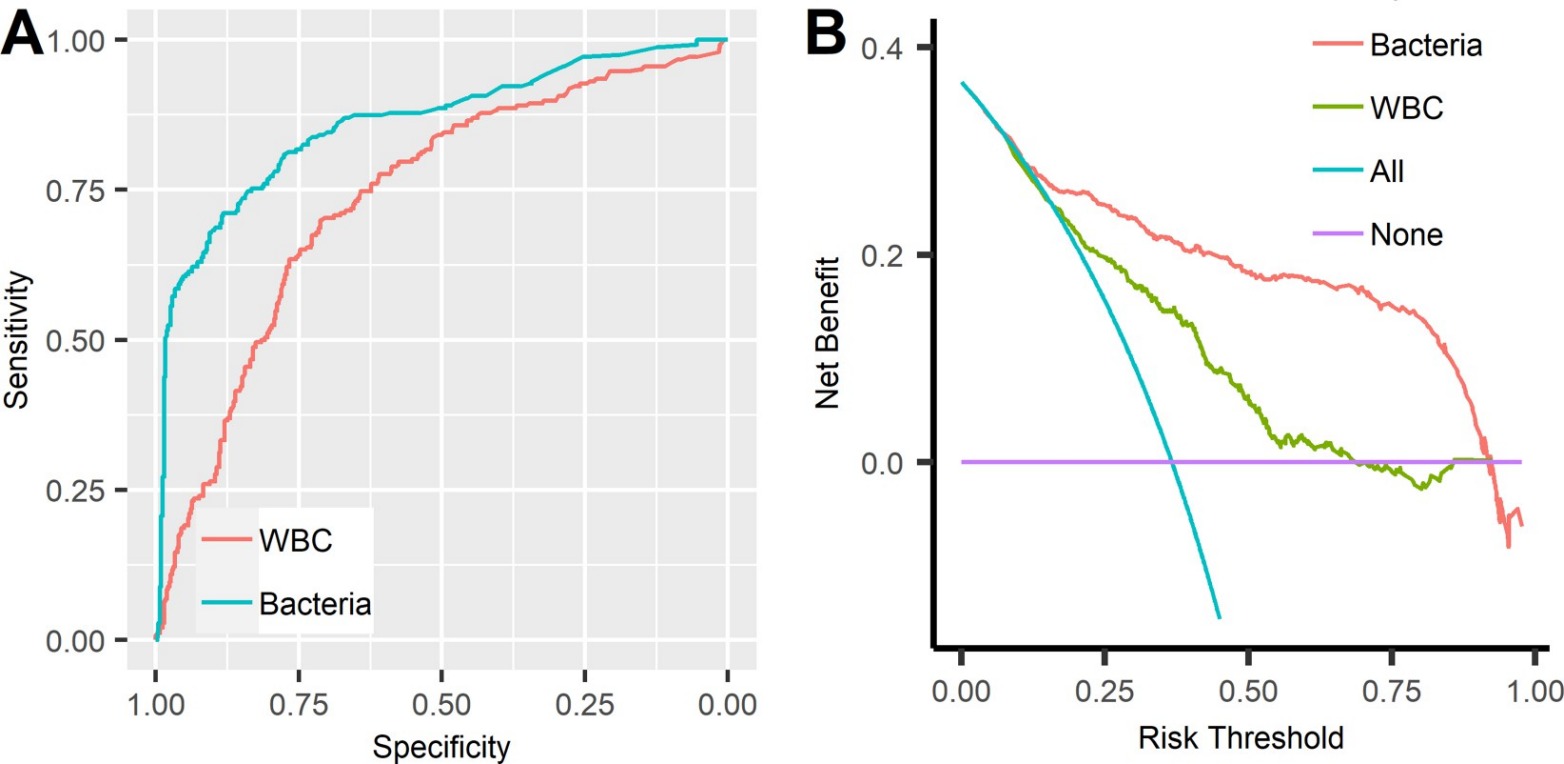
The receiver operating and decision curves of bacteria and WBC. A, receiver operating curve; B, decision curve. (A) ROC curves of the urine parameters for UTI screening; (B) decision curves of the urine parameters for UTI screening.

**Fig 1** shows the ROC curves **(Fig 1A)** and decision curves **(Fig 1B)** of the urine parameters for UTI screening. The AUCs of WBC and bacteria were 0.74 (95% CI: 0.70–0.78) and 0.86 (95% CI: 0.83–0.89), respectively. The AUC of bacteria was significantly higher than that of WBC (*p*<0.001), indicating that the screening performance of bacteria is higher than that of WBC. Furthermore, the bacterial decision curve was above the WBC curve **(Fig 1B)**, indicating that the net benefit of bacteria is higher than that of WBC.

In addition, we searched the PubMed database and found that four studies investigated the performance of WBC and bacterial counts, which were determined by a Sysmex UF-5000, for UTI screening. As shown in **Table 2**, the AUCs of WBC and bacteria in this study seem lower than those in previous studies. In addition, both our study and previous studies found that the AUC of bacteria was higher than that of WBC.

**Table 2:** Studies investigating the screening performances of WBCs and bacteria (determined by Sysmex UF-5000).

Author	Year	UTI/Non-UTI	AUCs	
			Bacteria	WBC
**Kim [7]**	2018	336/1094	0.95	0.84
**De Rosa [11]**	2018	797/1922	0.97	0.84
**Ren [16]**	2018	93/498	0.96	0.77
**Enko [10]**	2021	98/246	0.94	0.86
**The present study**	2021	246/425	0.86	0.74

### A nomogram with urine bacteria, nitrate, and WBC for screening UTI

**Fig 2:**
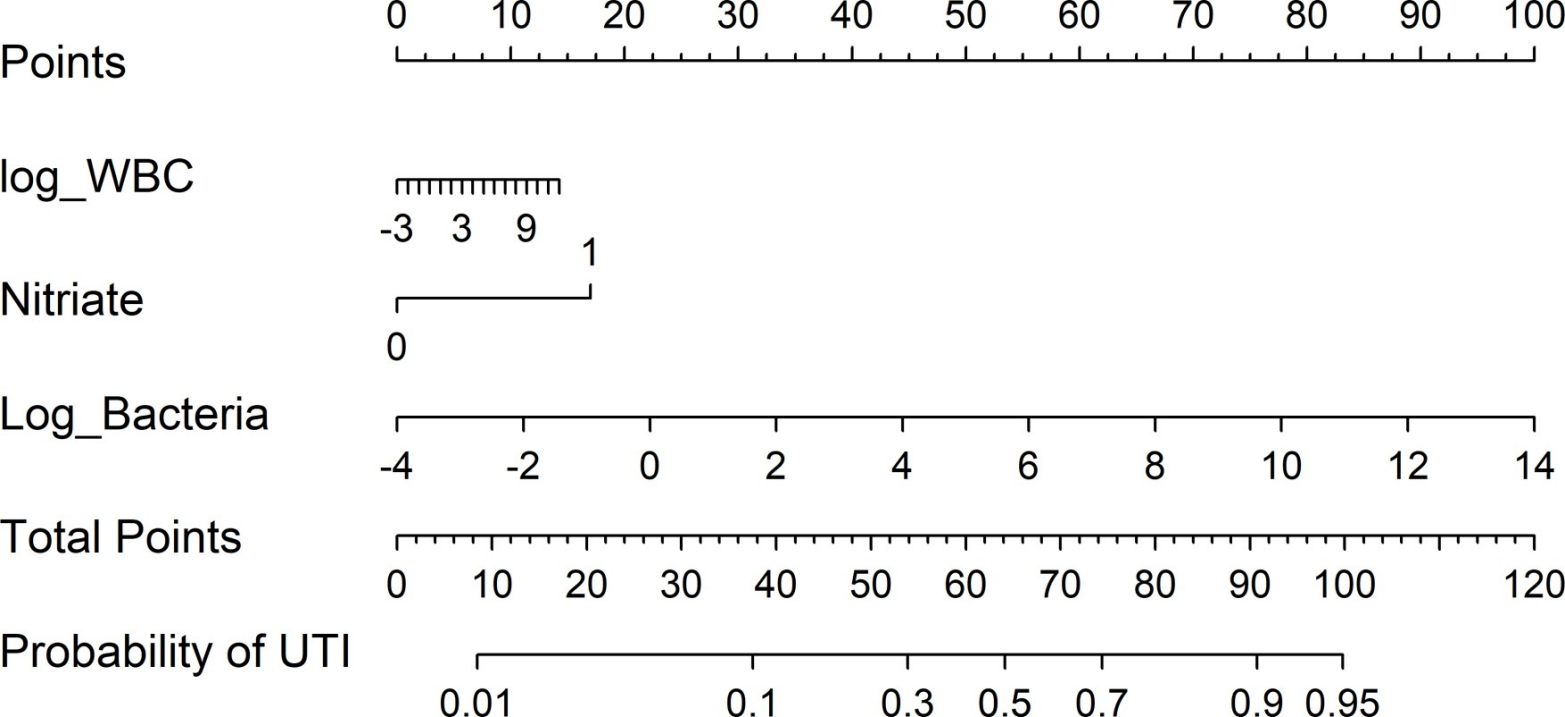
A nomogram for screening UTI.

**Fig 2** shows a nomogram for UTI screening. This nomogram was based on three common urine parameters: nitrate, bacteria, and WBC. Among these urine parameters, bacteria had the strongest effect on the probability of UTI, followed by nitrate, while WBC had the weakest effect. The calibration plot of the nomogram is presented in **S1 Fig**. Generally, the calibration plot presented excellent agreement between the actual observation and nomogram prediction.

### A decision tree for UTI screening

**Fig 3:**
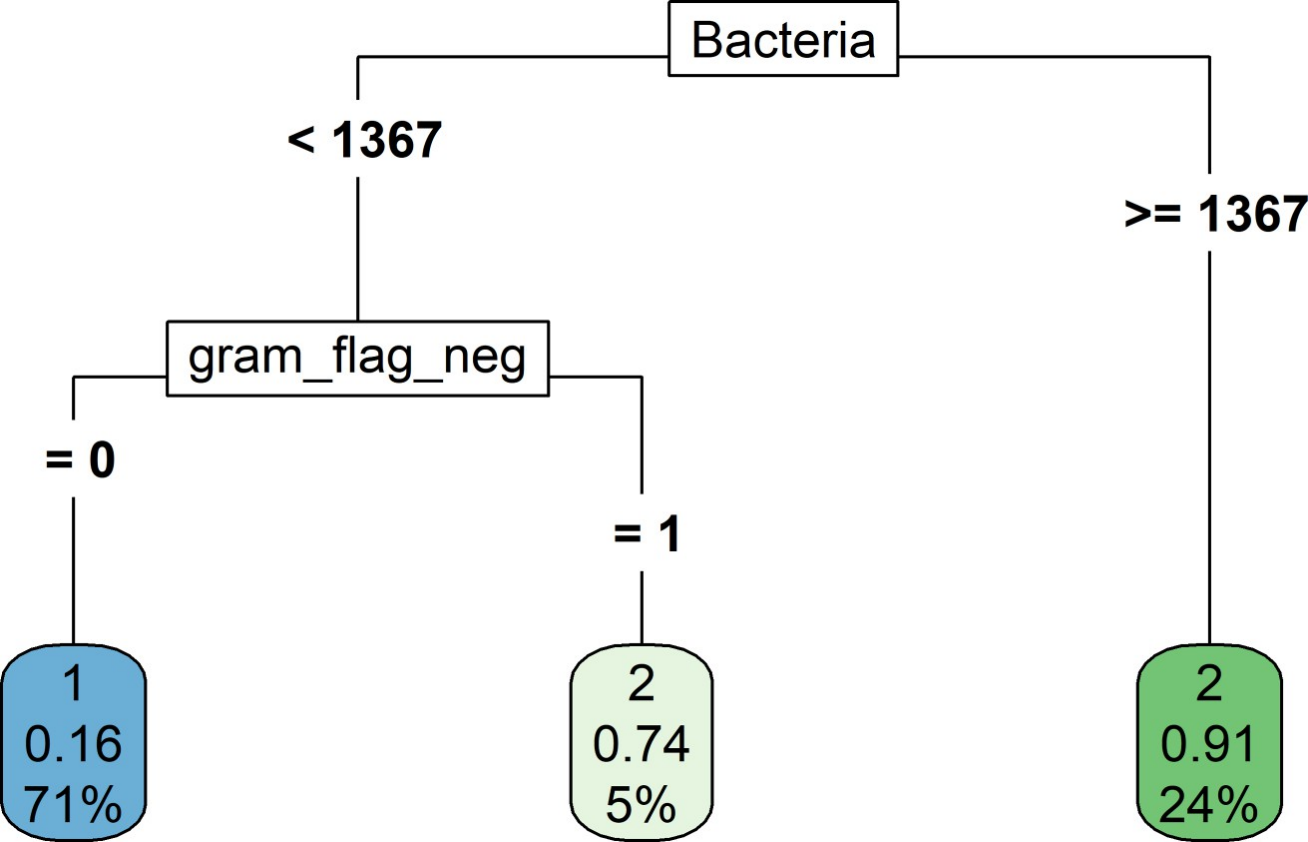
A decision tree for UTI screening.

**Fig 3** shows a decision tree for UTI screening. In the initial step, we included nitrite, bacteria, WBC, the Gram flag, and the UTI flag in the tree model and found that the tree had the highest accuracy when the number of splits (nsplit) was 2, indicating that the tree with two splits had the highest diagnostic accuracy. Therefore, only bacteria and the Gram flag were included in the tree. Notably, patients with urine bacteria < 1367 /μl had a low probability of UTI, except for patients with the Gram-negative flag, for whom the probability of UTI was 74%. The sensitivity and specificity of this decision tree were 0.69 (170/246) and 0.95 (403/425), respectively. In contrast, when the specificities of WBC and bacteria were fixed at 0.95, their sensitivities were 0.19 and 0.60, respectively.

### Accuracy of the Gram flag of the UF-5000 for discriminating gram-positive and gram-negative bacteria in urine culture

Among all participants, 158 patients had a high probability of UTI, with urine bacteria >1367/μl. As shown in **Table 3**, 7 of 10 gram-positive bacteria-infected patients were predicted by the UF-5000, and 106 of 131 gram-negative bacteria-infected patients were predicted by the UF-5000. Therefore, the sensitivities of gram-positive and gram-negative flags in patients with high probability of UTI (bacteria >1367/μl) were 0.70 (7/10) and 0.81 (106/131), respectively. However, the specificities of gram-positive and gram-negative flags were 0.78 (116/148) and 0.70 (19/27), reppectively. The positive predictive value (PPV) of gram-negative and gram-positive flags were 0.93 (106/114) and 0.18 (7/39), respectively.

**Table 3:** The agreement between Sysmex UF-5000’s Gram flag and urine culture results.

Urine culture	Sysmex UF-5000 Gram Flag			Total
	Gram-negative	Gram-positive	Unclassified	
**Gram-negative**	106	22	3	131
**Gram-positive**	3	7	0	10
**Fungus or others**	0	4	0	4
**Mixed infection**	5	6	2	13

## Discussion

The major findings of this study were as follows: First, the WBC and bacteria determined by the Sysmex UF-5000 had high screening performance for UTI. The screening performance of bacteria was higher than that of WBC. Second, a routine urine parameter-based nomogram and decision tree can be used to screen UTI. The decision tree can improve the screening performance of urine parameters. Third, the Gram flag of the UF-5000 has moderate accuracy for discriminating gram-positive and gram-negative bacteria, with sensitivities between 0.70 and 0.80.

Our study supports some findings of previous studies. For example, the screening performance of bacteria was higher than that of WBC [3, 4], and combining WBC and bacteria can improve the UTI screening performance [3]. However, this study has some novel findings. One is the nomogram we constructed for UTI screening; this nomogram has high screening performance, as indicated by the calibration plot. Notably, the effect of WBC and nitriates on the screening performance was slight, indicating that WBC can slightly improve the screening performance of bacteria. This finding was also supported by the decision tree, in which only bacteria and the Gram flag alone can be used to initially discriminate UTI and non-UTI. To the best of our knowledge, no study has employed a decision tree to assess the screening performance of WBC and bacteria. The decision tree had a sensitivity of 0.69 and specificity of 0.95. In comparison, the sensitivities of WBC and bacteria were lower than that of the decision tree when the specificity was fixed at 0.95. This result suggests that the decision tree represents a useful method for improving the screening performance of urine parameters.

Another novel finding is that the Gram flag had a modertate accuracy for predicting gram-negative or gram-positive bacteria infection in patients with high probability of UTI (bacteria >1367/μl). In these patients, the probability of UTI was 92% (145/158). Because 72% of UTI patients were infected by gram-negative bacteria, the flag of Gram-negative has a high PPV of 0.93, indicating that in patients with bacteria >1367/μl and a flag of gram-negative, the probability of gram-negative bacteria infection is 93%. Under such a condition, antibiotics against gram-negative bacteria may benefit patients. However, the PPV for the flag of Gram-positive was only 0.18. The low PPV does not support using antibiotics against gram-positive patients in these patients.

The present study has some limitations. The major limitation of this study is the monocenter design and small sample size; therefore, multicenter studies with large sample sizes are needed to validate the present findings.

## Conclusions

Overall, this study indicates that the urine bacterial and WBC counts determined by the Sysmex UF-5000 analyzer have high screening performance for UTI. The decision tree can be used to improve the screening performance of routine urine parameters. The Gram flag of the Sysmex UF-5000 can be used to assist antibiotic choice in patients with high UTI probability.

## Supporting information

S1 Fig The calibration plot of the nomogram for screening UTI. (TIFF)
