# Supplementary figures and images for: Accuracy of the Sysmex UF-5000 analyzer for urinary tract infection screening and pathogen classification
Source: PLoS One. 2023 Feb 1;18(2):e0281118. doi: 10.1371/journal.pone.0281118 (PMC9891513; doi:10.1371/journal.pone.0281118)

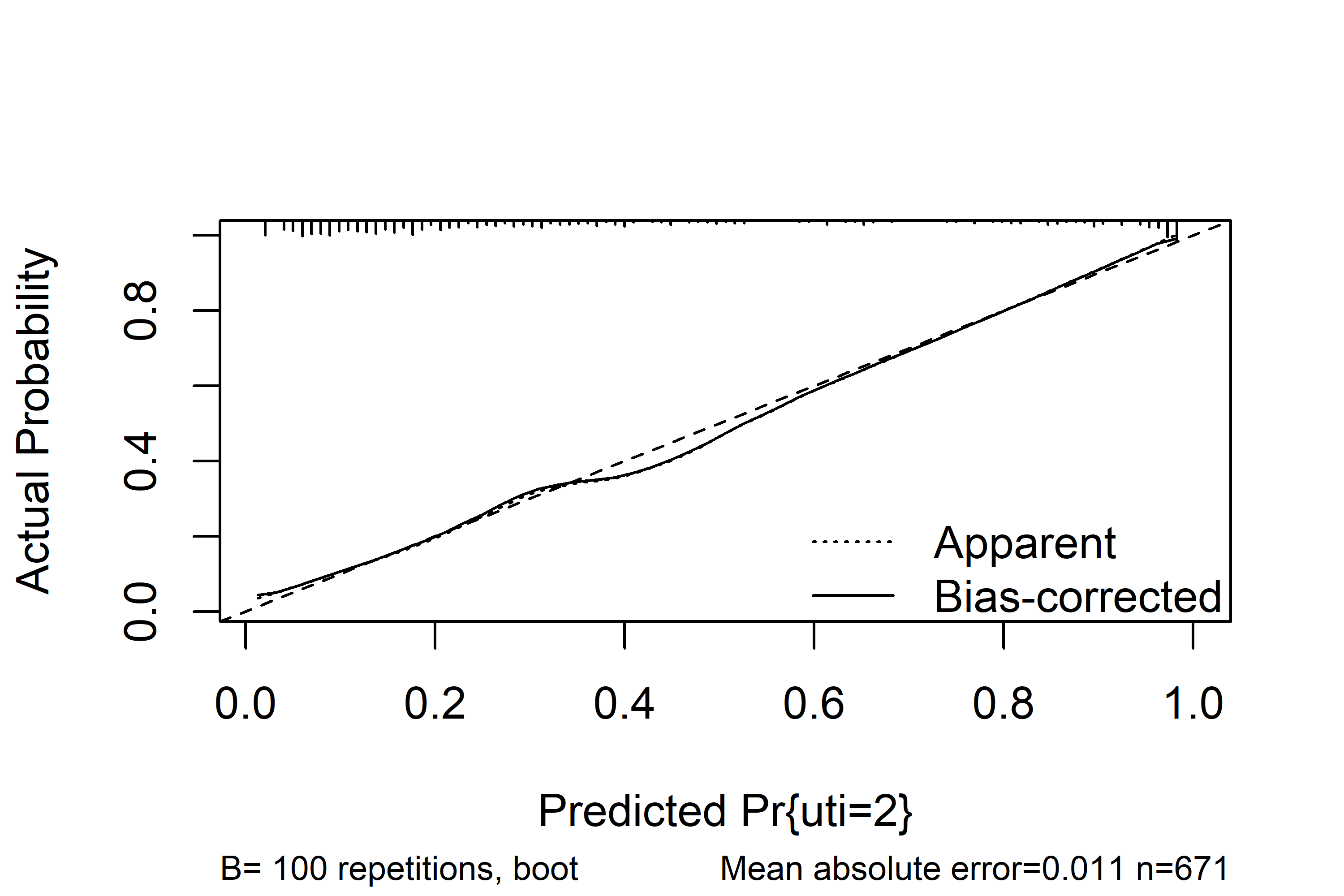

Supplement: S1 Fig — (TIFF) [file pone.0281118.s001.tiff]
